# Polydrug use during chemsex: single and intersecting sexual effects of commonly used drugs

**DOI:** 10.3389/fpubh.2025.1618070

**Published:** 2025-07-10

**Authors:** Tom Platteau, Jochen Schrooten, Corinne Herrijgers, Chantal den Daas, Mireia Ventura, Carol Strong, John de Wit

**Affiliations:** ^1^Department of Clinical Sciences, Institute of Tropical Medicine, Antwerp, Belgium; ^2^VAD, Brussels, Belgium; ^3^Institute of Applied Health Sciences, University of Aberdeen, Aberdeen, United Kingdom; ^4^Energy Control, Barcelona, Spain; ^5^Department of Public Health, College of Medicine, National Cheng Kung University, Tainan, Taiwan; ^6^Department of Interdisciplinary Social Science, Utrecht University, Utrecht, Netherlands

**Keywords:** sexualized drug use, chemsex, polydrug use, sexual effects, care and support

## Abstract

Chemsex refers to the intentional use of psychoactive substances to enhance, explore, or alter sexual experiences. The use of geolocation apps, and its link with physical, psychological, and social harms for the individual as well as society has been described ‘Polydrug use’—combining multiple substances to amplify or diversify effects—is frequently reported across these groups. This article provides a comprehensive overview of the sexual effects of substances commonly used in chemsex contexts. We introduce the Pharmacosex Wheel, a visual tool that categorizes drugs based on their pharmacological properties and their specific effects on sexual experience. Substances are grouped into seven classes: stimulants, empathogens, psychedelics, dissociatives, depressants, opioids, and cannabinoids. Each class is discussed in relation to its typical impact on sexual behavior, desire, sensation, and connection. To further illuminate the dynamics of polydrug use, we present a layered model that maps core and intersecting effects of combined substances. Core effects include stimulation (e.g., from stimulants), disinhibition (e.g., from depressants and dissociatives), and altered sensory perception (e.g., from empathogens, cannabinoids, and psychedelics). When substances with different core effects are combined, intersecting effects emerge—such as euphoria, empathy, or chill—which often result in rich and multifaceted sexual experiences. This model also incorporates user motivations, acknowledging that the choice of substances often aligns with specific desires or anticipated outcomes. Finally, we position the most commonly used substances within this framework, grouping them according to their chemical structure, pharmacological action, and the experiential reports of users. By approaching chemsex from a drug-positive perspective, without neglecting potential negative impacts, this article aims to foster a nuanced understanding of substance use in sexual contexts. Our goal is to support more informed research, destigmatize user experiences, and contribute to the development of tailored care within drug and sexual health services.

## 1 Background

The most commonly reported desired effects of sex under the influence of drugs include enhanced emotional connection, intense bodily sensations, disinhibition, and increased lust (1). The most studied form of sexualized drug use is *chemsex*, a term specifically describing the intentional use of particular drugs to intensify sexual experiences, primarily among gay, bisexual, and other men who have sex with men (gbMSM). Additional characteristics of chemsex include the use of geolocation apps, and its link with a range of physical, psychological, and social harms from both individual and societal perspectives (2). Chemsex has become a significant public health issue that warrants attention (3). Initially, the term chemsex was defined by the use of mephedrone (4-MMC), methamphetamine (crystal meth), *γ*-hydroxybutyrate (GHB), and γ-butyrolactone (GBL) (4). However, more recent studies have suggested a broader understanding of chemsex, with modified definitions and revised categorizations of substances (2, 5) based on regional differences (6, 7) and variations in user populations (8). To capture this variation, the term ‘pharmacosex’ was coined as a way to describe how wider populations experiment with a range of illicit substances that modify and enhance their sex lives (1).

As prevalence of chemsex differs depending on the used definition, it is hard to assess a reliable prevalence. Data from the EMIS-study in 2017 revealed lifetime prevalence of 15, and 10% in the past 12 months (9). A recent meta-analysis and review article estimates the prevalence of chemsex among gbMSM in Europe at 16% (95% CI: 11–21%) (5). However, higher prevalence rates have been observed in specific subpopulations. For example, 39.8 and 63% of gbMSM using pre-exposure prophylaxis (PrEP) for HIV prevention in France (10) and Spain (11) respectively reported engaging in chemsex. Additionally, 30.6% of users of gay dating platforms (12), 41% of men who have sex with men (MSM) attending sexually transmitted infection (STI) clinics (13), 36.1% of HIV-negative MSM (14), and 48.5% of gbMSM living with HIV (15) reported similar behaviors.

The most recent Drug Report by the European Monitoring Center for Drugs and Drug Addiction highlights an increase in polydrug use (16). Polydrug use refers to the simultaneous or sequential use of two or more psychoactive substances, either by deliberately combining different drugs or by using substances that contain multiple active ingredients (16). A specific increase in polydrug use for sexual purposes has been observed among gbMSM engaging in chemsex (17–20). Individuals often combine various substances either to mitigate negative side effects or to enhance their sexual experience through synergistic effects. Therefore, polydrug use in a sexual context is conceptually part of *chemsex*.

Much of the existing scientific research focuses on the effects of single substances or the combined effects of two substances, such as MDMA and GHB (21), methamphetamine and cannabis (22), GHB and 3-MMC (23), or alcohol and cocaine (24). However, these studies typically do not examine the impact on sexual behavior or sexual function.

Polydrug use has been linked to more pronounced physical and psychological effects (25), but there remains a significant gap in understanding the reasons behind substance combinations, the specific substances involved, and the resultant effects experienced by users (26).

The aim of this paper is to provide a comprehensive overview, from a drug-positive perspective, of key aspects of the phenomena of *chemsex*. This includes exploring the contexts in which these terms are used, their meanings, how drugs used during sex can be classified, the specific substances and combinations involved, their individual and intersecting effects, and the motivations behind their use for sexual purposes.

While several studies have reviewed the substances used in sexual contexts, there remains a significant gap in comprehensive information regarding the specific sexual effects of the most commonly used drugs before and during sexual encounters. To address this gap, we utilize the established framework of the *Drugs Wheel* (27) to categorize various substances and present the most frequently reported sexual effects, with a focus on how these drugs impact the sexual experience. Additionally, this paper introduces the *Pharmacosex Wheel*, a visual tool that categorizes drugs based on their individual effects on sexual experiences, providing a clearer understanding of the relationship between drug use and sexual behavior.

Building on the single-effect overview, we introduce a visual model illustrating the intersecting effects of substances on users’ sexual experiences, referred to as the *Layered Model of Core and Intersecting Drug Effects*. This model not only highlights the diverse impacts of drug use on sexual experiences but also captures the complex motivations driving *chemsex*.

While this overview is not exhaustive—due to the complexity of the phenomenon and the overlapping effects of multiple substances—it serves as a useful framework for clinicians and researchers encountering this behavior. It offers a broad understanding of the phenomenon, helping to inform further inquiry and intervention. We believe that both the *Pharmacosex Wheel* and the *Layered Model of Core and Intersecting Drug Effects* provide valuable insights and are essential for developing targeted interventions that address the unique needs of different user populations.

## 2 Classification and effect of drugs that are used before and during sex

In recent years, the development of new substances, collectively referred to as *New Psychoactive Substances* (NPS), has surged (28). This term encompasses a broad range of synthetic and plant-derived psychoactive substances that have gained prominence since 2008 (27). NPS include newly designed synthetic drugs, plant-based compounds or their extracts, previously existing substances that have recently entered the market, and certain pharmaceuticals or medications. The number of identified NPS has risen dramatically, growing from 236 in 2012 (29) to 930 in 2023 (30). A specific category of NPS, known as *cathinones* (e.g., *mephedrone* (4-MMC), *metaphedrone* (3-MMC), and others), is commonly used in sexual contexts (2).

To capture the diverse effects of newly developed substances, a new categorization was deemed necessary. Building on the work of Leonard (31), Adley and colleagues (27) developed the *Drugs Wheel*, a taxonomy that includes both established drugs and NPS. The primary goal of the *Drugs Wheel* is to offer a comprehensive framework, integrating information on the effects of individual drugs, their actions on receptor pathways, and harm reduction strategies for each of its seven categories (27).

**Figure 1:**
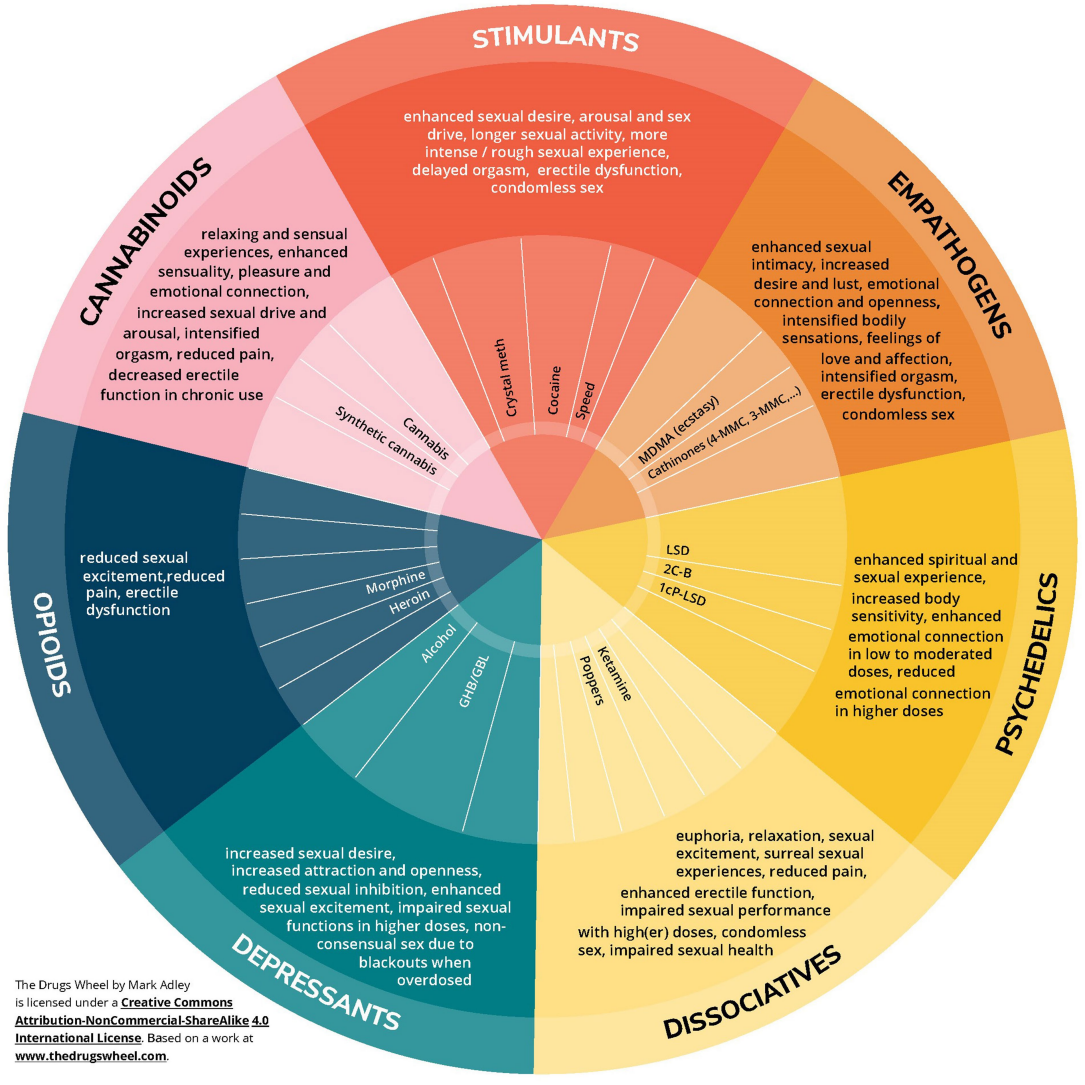
Pharmacosex wheel.

This *Drugs Wheel*, created in collaboration with stakeholders and end-users (27), organizes substances into seven categories: stimulants, empathogens, psychedelics, dissociatives, depressants, opioids, and cannabinoids. With the approval of the original developers, Schrooten and colleagues adapted this taxonomy into the *Pharmacosex Wheel*, published in Dutch (32). Figure 1 below presents a translation of this *Pharmacosex Wheel* (32) and provides an overview of the sexual effects of individual drugs, including their impact on sexual desire, arousal, and orgasm, within each of the seven categories outlined in the *Drugs Wheel* (27).

While the *Pharmacosex Wheel* offers a general overview, it is important to recognize that the effects experienced from drug use can vary significantly due to individual differences and personal motivations. Clinical experience indicates that the specific drugs a person chooses often depend on the desired outcome. Users may seek particular aspects of sexual functioning or experiences and select their substances accordingly. For example, one person may use a dissociative drug to minimize physical discomfort during sexual activity, while another might prefer an empathogen to enhance emotional connection with their partner.

Drugs within the same category often share similar effects, though these effects are not always identical. The categorization of substances in the *Drugs Wheel* is primarily based on their chemical structure, rather than their subjective effects. As a result, some drugs may be classified within one category, yet produce effects that overlap with substances in another. This overlap is evident in their usage. For example, certain cathinones (e.g., 3-MMC, 4-MMC), initially categorized as stimulants, also exhibit empathogenic effects (as noted in the latest version of the *Drugs Wheel*), making them particularly appealing for use in sexual contexts.

In the following sections, we will explore each drug category in greater detail, examining its specific effects on sexual experiences and behavior.

### 2.1 Stimulants

Commonly reported stimulants used in sexual contexts include crystallized methamphetamine (“crystal meth” or “TINA”), cocaine (“coke”), and amphetamine (“speed”). These stimulants primarily affect the dopamine reward center, inducing euphoria and heightened sexual arousal, which generally enhances the pleasurable experience of sex (33). As a result, stimulants typically increase sexual desire (34, 35).

Cocaine, in particular, has been linked to both increased and inhibited sexual desire. Initially, many men using cocaine experience heightened sexual arousal. However, with prolonged use, cocaine can negatively impact sexual functioning, diminishing desire, impairing erectile function, and affecting orgasm (36).

Stimulants are also known to prolong sexual activity, primarily due to their energizing effects and their ability to delay male orgasm (34). While this delay is a side effect, some individuals view it as beneficial in a sexual context. However, a common and undesirable side effect of stimulant use is erectile dysfunction (34, 37). Research has shown that the strength of the stimulant is directly related to the difficulty in achieving or maintaining an erection (38). As a result, stimulants are often combined with erection-enhancing medications like Kamagra or Viagra in sexual settings, a practice reported by 53% of chemsex users (39). These medications, however, are not included in the *Drugs Wheel* as they do not have psychoactive effects.

Sexual experiences under the influence of stimulants can become more intense or even rougher, which may increase the likelihood of engaging in risky sexual behaviors. This risk manifests in two ways: through the heightened intensity of sexual activity and the physical abrasions that can result from rough sex. While much of the research has focused on the LGBTQIA+ community (40), similar behaviors have also been observed in heterosexual men (38). Despite the perception that stimulants enhance sexual experiences, the long-term combination of stimulants and sexual activity can lead to negative outcomes, including significant impacts on one’s social life, as well as mental and physical health (41).

### 2.2 Empathogens

MDMA, the active ingredient in ecstasy, is a well-known empathogen often referred to as “the love drug.” It is commonly used to enhance sexual intimacy, desire, and overall sexual experience (33). Among empathogens, cathinones are also widely used in sexual contexts due to their ability to increase sexual desire (2). Substances like 4-MMC (mephedrone) and 3-MMC (metaphedrone) are frequently associated with chemsex activities (42) but are increasingly reported by individuals across a range of sexual and gender identities (43, 44). Cathinones combine both stimulant and empathogenic effects, which is why they were recently moved from the stimulant category to the empathogen category in the latest version of the *Drugs Wheel* (45).

Empathogens are known to enhance feelings of empathy, foster emotional connection, and promote emotional openness. These substances intensify bodily sensations, facilitate deep and open communication, and often lead sexual partners to experience feelings of love and affection (46). In particular, empathogens are commonly associated with heightened sensual experiences, emotional depth, and stronger feelings of love and intimacy. Women, in particular, report increased sexual experiences, including enhanced desire, lust, and more intense orgasms (46).

However, MDMA’s use is also linked to erectile dysfunction in a significant proportion of men—up to 40%—although this is not always perceived as problematic. For some, it allows for a shift away from orgasm-driven sex and encourages experimentation with more sensual, less goal-oriented sexual experiences (1, 47).

Moreover, sexual risk behaviors, such as having multiple partners or engaging in condomless sex, are often reported by individuals who have sex under the influence of MDMA (46). These findings suggest that while MDMA can enhance emotional and sexual connection, it also carries significant risks, particularly regarding sexual health and safety.

### 2.3 Psychedelics

Psychedelics are generally known to induce spiritual experiences and euphoria (27). Recently, a variety of new hallucinogenic substances have emerged on the market. One such substance is 1cp-LSD, a prodrug that, once ingested, is converted into LSD in the body (48). More recently, phenethylamines like 2C-B have gained popularity for their use in enhancing sexual experiences. Alexander Shulgin, who developed 2C-B, described it as the most erotically stimulating substance among the nearly 200 drugs he synthesized (49). Pharmacologically, 2C-B shares similarities with MDMA (an empathogen), combining the effects of classical psychedelics—such as dissociation, perceptual changes, and creativity—with feelings of euphoria, emotional openness, happiness, and positive affect (50). Research suggests that in terms of sexuality, 2C-B induces pleasant euphoria, fosters strong emotional connections, and heightens body sensitivity (51).

Psychedelic substances tend to influence sexual experiences most significantly at low to moderate doses (52, 53). However, when taken in higher doses, engaging in sexual activity can become more challenging, as each partner may become immersed in their own “trip,” leading to a notable reduction in both mental and physical connection. Consequently, the *set* (mindset and emotional state) and *setting* (context of use) are crucial factors in shaping the experience of a hallucinogenic trip, especially in the context of pharmacosex.

Despite their potent effects on sexual experience, the use of psychedelics—particularly 2C-B—remains relatively limited (39). While these substances can significantly alter sexual dynamics, their impact is highly dose-dependent, and their use in sexual contexts requires careful consideration of the user’s mindset and environment.

### 2.4 Cannabinoids

Cannabis is a versatile substance, and when used in sexual contexts, it is primarily sought for its relaxing and sensual effects (27). The impact of cannabis on sexual experiences can vary depending on several factors, including its origin (natural versus synthetic), strain (higher THC versus higher CBD), potency (THC percentage), dosage, method of consumption (e.g., smoking, edibles, vaporizing), and the individual’s tolerance.

Historically, cannabis has been regarded as an aphrodisiac, but more recent research on its effects on sexual function is mixed. While cannabis use is often reported as pleasurable and satisfying, chronic use may lead to a decrease in testosterone levels and negatively affect male erectile function (33). Additionally, the sexual effects of cannabis can be either positive or negative, influenced by the factors mentioned above (54). Positive experiences may include enhanced sensuality, pleasure, and emotional connection, while negative effects can include nausea, confusion, and paranoia. Generally, lower doses of milder cannabis strains tend to carry fewer risks of unwanted side effects.

Many women report experiencing increased sexual desire, better orgasms, and reduced pain during sex when using cannabis (54). Men also report heightened arousal and more intense orgasms (55). A Canadian study found that cannabis use can help maximize pleasure and social connection with sexual partners, while simultaneously reducing anxiety and shyness (56). These findings suggest that cannabis may have the potential to enhance both the physical and emotional aspects of sexual experiences, though its effects can vary significantly depending on the specific use and individual factors.

### 2.5 Depressants

The most commonly reported depressants used before and during sex include alcohol, GHB, and GBL (“G”), all of which share sedative effects, along with a sense of increased confidence and sociability (27). Alcohol, the oldest known drug, has a long-standing association with sexual activity, dating back to the earliest records of human history (57). Its widespread legal status and availability make it a commonly used substance in sexual contexts.

While alcohol is often thought to enhance the sexual experience, its effects are more nuanced. After consuming one or more alcoholic drinks, both men and women commonly experience increased sexual desire and heightened self-confidence (58), as social inhibitions are lowered due to alcohol’s depressant effects (59). In low doses, alcohol can increase testosterone levels in women, which may enhance sexual desire and intensify orgasms (59). However, in higher doses, alcohol can reduce sexual desire and impair sexual performance among men and women. Men may find it more difficult to achieve or maintain an erection, and may face challenges with ejaculation (60).

GHB and its prodrug GBL (which is converted into GHB in the body after ingestion) are widely used depressants in the class of NPS. Initially popular among gbMSM, their use is now increasingly reported among heterosexual men and women. These substances are typically used to enhance sexual excitement, increase attraction, and promote a sense of sexual openness (61).

Despite their desired effects, GHB and GBL are associated with significant health risks. Notably, a quarter of participants in a study of 60 individuals reported experiencing blackouts, which can lead to risky sexual behavior and boundary violations (62). Both GHB and, particularly, the more potent GBL, are difficult to dose accurately, which contributes to these dangers. To minimize the risk of blackouts and enhance pleasure, these substances are often combined with mid- to long-acting stimulants. Harm reduction strategies, such as using a pipette for precise dosing and keeping track of the timing of doses, are essential to reduce the risk of adverse effects and ensure a safer experience.

### 2.6 Dissociatives

Dissociatives are commonly used in sexual contexts to induce euphoria and relaxation (27). The most frequently used dissociatives in sexual settings are amyl- and butyl-nitrates (“poppers”) and ketamine (“keta,” “special K”). While both substances share similar effects, their impact can vary significantly depending on the substance and dosage.

Poppers (amyl/butyl-nitrates) are inhaled as a gas and produce a brief, intense feeling of intoxication. The effects include mental and physical relaxation, along with an enhancement of erectile function (33). Poppers are particularly popular in gbMSM communities, as they can make anal sex more comfortable and less painful. However, when combined with erection-enhancing drugs, poppers can be dangerous due to their blood pressure-lowering effects, which can lead to serious health risks (63).

Ketamine, another dissociative commonly used in sexual contexts, affects users differently depending on the dose. At low doses, ketamine can increase sexual desire, while higher doses may reduce this desire by impairing coordination and concentration. Users who take higher doses often report dizziness, nausea (64), and a sense of detachment from their bodies due to ketamine’s dissociative properties. Higher doses of ketamine can make the sexual experience feel surreal. Very high doses of ketamine provoke deep dissociation and can lead to what is colloquially known as a “K-hole,” where the individual feels as if they are completely disconnected from their physical surroundings. Ketamine is also a potent analgesic, meaning it can reduce pain, potentially enabling rougher sexual activity (65). However, this increased tolerance for physical discomfort can raise the risk of engaging in high-risk sexual behaviors, such as condomless sex, and fisting (66), and thereby heightens the potential for acquiring sexually transmitted infections (67), including HIV.

### 2.7 Opioids

When opioids are first used, they can lead to initial effects such as delayed ejaculation in men and improvements in vaginismus in women, which may create a perception of enhanced sexual function. However, prolonged use of opioids (e.g., morphine, heroin) is generally associated with negative effects on sexual health, including a significant reduction in sexual desire and an increase in erectile dysfunction (33). Due to their primarily detrimental impact on sexual function, opioids are rarely used in recreational contexts such as chemsex. As a result, opioids are seldom discussed in the literature on these phenomenon, and their role in sexual behavior is not explored further in this article.

## 3 Intersecting effects

As mentioned earlier, polydrug use is common, and in some cases, the intersecting effects of combining two or more substances can significantly influence sexual experiences. In other instances, however, the effects may stem from a single substance in the combination, such as the empathogenic effects of MDMA when paired with a stimulant. Thus, the sexual effects observed may be either the result of intersecting effects from the combination or the individual effects of each substance. Additionally, the impact of some substances can vary depending on dosage. For example, certain substances may enhance sexual desire at lower doses but have the opposite effect at higher doses.

Our primary focus is on the intersecting sexual effects of substances used together before and during sex. While we recognize that individuals often combine substances from different categories in sexual contexts, the categories outlined below have been the most frequently reported in both research focused on chemsex (2) and broader studies on sexualized drug use, as discussed in the previous section.

**Figure 2:**
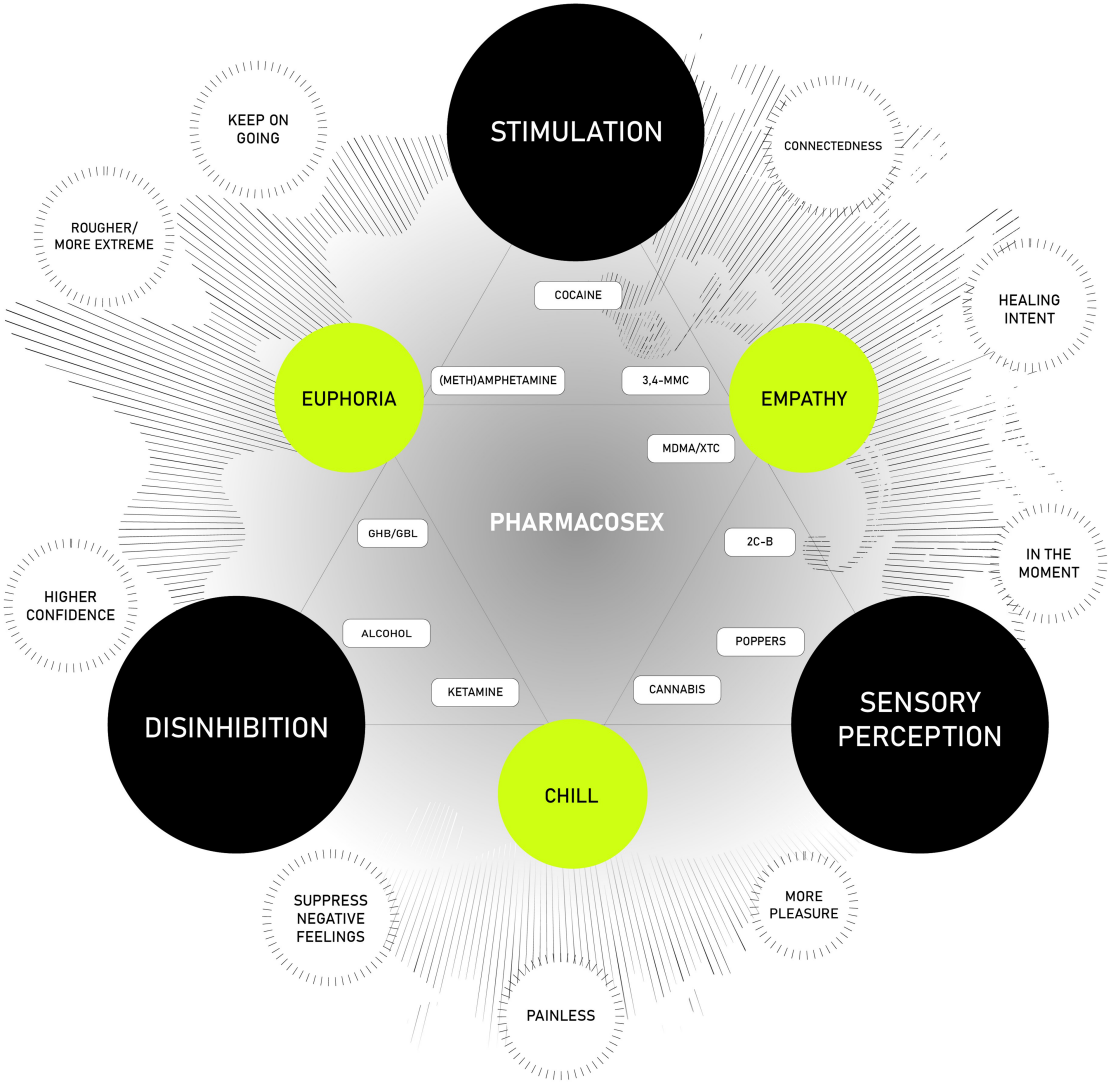
Layered model of core and intersecting drug effects.

People combine substances because they seek certain effects. However, little is known about these combined effects. That is why we consulted various experts for an ‘expert consultation’. These experts approach the topic from their different areas of expertise (pharmacology, sexual health, psychology, criminology, and sociology) to gain more insight into the motives, specific substances, and combined effects of certain categories of substances. Resulting from this expert consultation, we provide a schematic overview of the most commonly reported intersecting sexual effects that result from engaging in pharmacosex (Figure 2). This figure should be interpreted as a tool to better understand individuals’ experiences when combining drugs in a sexual context, rather than a precise pharmacological or toxicological representation of the outcomes of these drug combinations. Our overview of intersecting drug effects, developed from the information we have gathered and supplemented with the experiences and expertise of the author group, is intended as a starting point for discussion. As such, the figure primarily serves as a prime for research and clinical practice.

The model is structured in layers: the black circles represent the ‘core’ effects of commonly used substances (‘uppers’, ‘downers’, ‘hallucinogens’), while the green circles highlight new labels that emerge from the combination of two core effects. The outer white circles represent the motivations for using these substances in a sexual context, derived from existing literature. At the center of the figure, we place the most commonly used substances taken before and during sex, organized according to their chemical structures and the effects users experience.

The black circles within the schematic represent the ‘core’ effects associated with the most commonly used categories of substances in a sexual context: stimulation (stimulants), disinhibition (depressants and dissociatives), and sensory perception (empathogens, cannabinoids, and psychedelics). Stimulation encompasses the arousal and excitation of both the body and mind in a sexual context, triggered by activities or stimuli that elicit sexual desire and response. This experience varies widely among individuals and often involves both physical and psychological elements.

When considering the use of substances to enhance sexual experiences, it is crucial to recognize that substances like stimulants and empathogens can produce both positive and negative effects. These include increased arousal, improved endurance, heightened sensitivity, and a potential intensification of the sexual experience. However, these effects can vary depending on the specific substance combination, dosage, and individual user characteristics.

We conceptualize disinhibition in a sexual context as the reduction or removal of barriers or inhibitions that typically govern sexual behavior. Its manifestations can vary widely among individuals, influenced by factors such as arousal, relaxation, confidence in a partner, and the presence of a communicative, non-judgmental environment. For many, disinhibition enables the free expression of sexual desires and fantasies, fostering open communication and potentially leading to a more fulfilling sexual relationship. In some cases, it may encourage individuals to explore more adventurous or riskier behaviors, broadening the scope of their sexual experiences. However, it is important to emphasize that while disinhibition can facilitate positive experiences, it should always be exercised within the boundaries of mutual consent and respect for all parties involved. On the back side, disinhibition holds the potential of negative impacts by causing a lack of judgment, especially if substances have been administered unknowingly or in high doses.

With sensory perception, we refer to a heightened desire or interest in sexual activity, driven by both physical and psychological factors. The use of psychedelics, empathogens, and cannabinoids can significantly alter perception, mood, and consciousness. Some individuals report intensified sensory experiences, a deeper emotional connection with their partners, and an enhanced sense of belonging when using these substances in a sexual context. Others may experience altered perceptions of time, increased connectedness, or heightened awareness of bodily sensations. These effects can lead to a more intimate and meaningful sexual experience for some individuals.

Between these core effects, the triangles in the model represent intersecting effects that arise from the interaction of different categories of substances (e.g., euphoria, empathy, chill). These intersections often produce complex and multi-faceted sexual experiences.

In a sexual context, euphoria refers to an intense state of pleasure, happiness, and satisfaction that is typically experienced during or following sexual activity. This euphoric state is often closely linked with sexual arousal and orgasm, contributing to an enhanced sense of emotional and physical pleasure. Empathy in sexual relationships refers to the capacity to understand and share the feelings of a partner, playing a key role in fostering healthy, consensual, and emotionally connected interactions. Sedatives and dissociatives, by inhibiting certain brain signals, can induce a state of emotional numbness and physical relaxation, leading to what some describe as a “chill” experience. However, in some cases, this can also result in a more profound state of sedation or dissociation from one’s surroundings, which may either enhance or diminish the overall sexual experience, depending on the individual and context.

Encircling the two triangles, we highlight the various motivations that individuals report for using these substances. These motivations reflect their aspirations for a fulfilling and enhanced sexual experience, as previously discussed. The choice of a particular drug is often driven by the desired effect, the substances available within an individual’s sexual network, and their access to and availability of specific substances. Insights into these motivations have been gathered from scientific literature and reviewed by the author group, which includes experts across diverse fields such as pharmacology, criminology, psychology, behavioral sciences, sociology, and sexual health. Although other motivations—not specifically related to sex—may apply for people (e.g., self-medication), we opt to limit the motivations that relate to the actual sexual experience.

At the center of the figure, we position the most commonly used substances based on their classification within the Drugs Wheel (27). These substances are grouped according to their chemical structure, pharmacological effects, and the experiences reported by individuals who use them (derived from clinical practice and firsthand accounts). Additionally, substances from other categories are included when their effects closely align with those identified in the intersecting effects model, acknowledging the complex nature of polydrug use and the overlaps between different drug categories.

## 4 Discussion

The initiation drug use before or during sexual activity is driven by a wide range of motivations, from hedonistic pursuits where drug use enhances the qualities valued in sexual experiences (68), to coping mechanisms for addressing daily life challenges such as stress, loneliness, or anxiety (69, 70). For some, the use of drugs in a sexual context aims to increase emotional connection, heighten body sensations, promote disinhibition, or intensify sexual desire (1). Additionally, for certain individuals, engaging in chemsex serves as a way to manage negative emotions or difficult life experiences, including loneliness, anxiety, traumatic stress, low self-esteem, or a way to survive (71). Loneliness, for example, may result from an accumulation of intersecting adverse experiences (72).

While many individuals manage their drug use during sex with self-control, preventing negative outcomes associated with chemsex (72), it is important to recognize that all sexualized drug use carries the potential for harmful consequences (72). Negative health outcomes linked to this behavior have been documented in various studies (73–75). These include drug-related health issues such as addiction, overdose (76, 77), and an increased risk of HIV and STIs (78, 79). Mental health challenges, such as depression, are also common, particularly among people who inject drugs (primarily methamphetamine and cathinones) during chemsex (‘slamming’) (80), though findings on this are not always consistent (15). Other concerns include sexual health impairments, particularly non-consensual sex (61, 81), and an increased number of deaths reported in certain regions (82–84). For example, over 40% of 1,549 chemsex participants across four European countries reported experiencing ‘unwanted side effects’ related to their chemsex use (81). Recognizing the growing prevalence of chemsex, the World Health Organization (WHO) in 2022 emphasized the urgent need for tailored strategies to address its impact (3).

From an individual perspective, there is often a discrepancy between the desired experience and the actual experience. We propose that the greater this discrepancy, the more likely individuals are to experience negative emotions when reflecting on their chemsex behavior. To help minimize this potential gap—and its negatively evaluated impact—a conceptual framework on self-control was developed. This framework includes four key components: goal setting, goal enactment, goal progress appraisal, and goal adjustment (68).

Despite the potential harms and negative experiences, many users find that the benefits of positive sexual experiences often outweigh the negative effects, with some individuals reporting no harm at all. However, adverse outcomes, including various negative health impacts, have still been widely documented (72). Other dynamics, may apply for the use of drugs in a transactional sex context, or a criminal context (e.g., spiking). As we utilize a drug-positive perspective, this is not the focus of this manuscript.

All substances, regardless of their category, have the potential to impair judgment and decision-making, which can lead individuals to make choices they might avoid when sober, potentially resulting in regret or unsafe situations. The combination of substances, especially in a sexual context, can also impact physical performance and arousal. While some individuals report increased confidence and reduced anxiety, excessive consumption can lead to sexual dysfunction and diminished performance, as well as several drug-related, physical and mental health issues, and even death, as described above. These risks are particularly important to consider when evaluating the use of sedatives and dissociatives in a sexual context.

Though the phenomenon of chemsex has been observed since the early 2000s, it was formally introduced in scientific literature since 2013 (75, 85). *Pharmacosex*, a term to describe sexualized drug use broader than chemsex, was coined even later, in 2021 (1). Within the broader *pharmacosex* umbrella, two additional but less extensively studied groups engage in substance use during sex. The first group includes men and women who participate in sex parties, commonly referred to as swingers. In this context, sexualized drug use is often labeled *‘WAP*,’ an acronym for Weekend Adult Party (32). The second group, known as psychonauts (86), comprises individuals who experiment with novel substances, intentionally dosing to enhance or tailor specific sexual experiences (32). Although these groups have received less research attention, the single and intersecting effects of sexualized drug use—central to the concept of *pharmacosex*—may similarly apply to their practices (32). Since its introduction in scientific literature, numerous studies have been published, primarily focusing on chemsex, with efforts centered on defining the phenomenon, identifying substances involved, exploring the context in which it occurs, and detailing associated harms.

Since 2019, research efforts have shifted from describing to understanding the phenomenon of chemsex with the aim of supporting individuals in achieving healthier sexual lives through harm reduction strategies. This work led to the development of a continuum model that outlines six phases individuals may experience on the path toward problematic chemsex (72). Additionally, a categorization system for substances used in sexual contexts was introduced, distinguishing three groups of substances: the most commonly reported or classic chemsex drugs (such as methamphetamine, GHB/GBL, mephedrone, and other cathinones like 3MMC, 2MMC, and 4MEC); substances occasionally linked to the chemsex scene (including ketamine, MDMA, and cocaine); and drugs frequently used alongside chemsex substances but not typically classified as such, including alkyl nitrites (poppers), cannabis, and erection-enhancing medications (2).

Parallel research introduced a conceptual framework emphasizing ‘self-control’ as a key element in reducing harm related to drug use in sexual contexts, including both chemsex and pharmacosex (68). This framework was subsequently integrated into a mobile application, Budd (73, 87–89).

We advocate for continued scientific debate on this topic. The goal is to improve the understanding of polydrug use and its individual and intersecting effects, with the ultimate aim of providing effective prevention and care for chemsex users.

The broader objective is to raise awareness among healthcare providers and peer support groups, enabling them to offer effective assistance to individuals seeking help. The outlined framework of single and intersecting effects on sexuality can serve as a valuable component of the broader “effective support puzzle” for addressing sexualized drug use. Ultimately, the aim is to integrate this support seamlessly into existing programs focused on drug and sexual health, ensuring comprehensive care for individuals engaged in chemsex.

## 5 Conclusion

The introduction of the Pharmacosex Wheel and the Layered Model of Core and Intersecting Drug Effects provide a visual framework that categorizes drugs based on their individual and combined effects on sexual experiences. This tool not only classifies substances but also captures the complex motivations driving chemsex behaviors. These insights are crucial for developing targeted interventions that address the specific needs of diverse user populations.

By enhancing the understanding of specific behavioral patterns associated with substance use in sexual contexts, the Pharmacosex Wheel can improve the effectiveness of educational materials, and the design of tailored treatment programs and prevention strategies. Furthermore, it can guide research efforts, focusing attention on under-explored aspects of sexualized drug use. The diverse applications of this tool underscore its importance in untangling the complex relationship between drug consumption and sexual health.

Through this article, we aim to expand the understanding of why individuals combine sex and drugs, moving beyond the typical substances often discussed in the literature. By providing an overview of the effects and offering deeper insights into the motivations behind chemsex, we seek to improve care for individuals who seek support from drug and sexual health services due to the negative impacts of their sexualized drug use.
